# Assessing Influence of Mismatch Repair Mutations on Survival in Patients After Resection of Pancreatic Ductal and Periampullary Adenocarcinoma

**DOI:** 10.3390/jcm13206185

**Published:** 2024-10-17

**Authors:** Elizabeth Prezioso, Eleanor Mancheski, Kylee Shivok, Zachary Kaplan, Wilbur Bowne, Aditi Jain, Harish Lavu, Charles J. Yeo, Avinoam Nevler

**Affiliations:** 1 Sidney Kimmel Medical College, Philadelphia, PA 19107, USA; elizabeth.prezioso@jefferson.edu (E.P.);; 2 Jefferson Pancreatic, Biliary and Related Cancer Center, Sidney Kimmel Cancer Center, Philadelphia, PA 19107, USA

**Keywords:** pancreatic cancer, pancreatic ductal adenocarcinoma, periampullary adenocarcinoma, mismatch repair, MMR, microsatellite instability, MSI, clinical outcomes

## Abstract

**Background**: Pancreatic ductal adenocarcinoma (PDAC) is the third leading cause of cancer-related deaths in the United States. Previous studies have indicated that microsatellite instability and deficient mismatch repair (MMR) may be associated with improved survival in patients with pancreatic cancer. Here, we aim to investigate the impact of deficient MMR (dMMR) status on oncologic outcomes in patients after resection of PDAC and periampullary adenocarcinoma. **Methods**: This is a single-institution, retrospective study based on a prospectively maintained database. Pancreatic ductal adenocarcinoma (N = 342) and periampullary adenocarcinoma patients (N = 76) who underwent pancreatic resection surgery between 2016 and 2021 were included. Immunohistochemistry staining results of MMR proteins and next-generation sequencing data were recorded. Cancer-type dependent Cox regression analyses were performed to assess overall and disease-free survival, which was complemented with a 1:2 propensity-score matching for each of the cancer types in order to compare oncologic outcomes. **Results**: A total of 418 pancreatic cancer patients were included in the analysis. Fifteen patients (3.5%) were diagnosed as dMMR (PDAC N = 7 and periampullary adenocarcinoma N = 8). Cox regression modeling of dMMR status interaction with TNM staging and cancer type revealed that dMMR status strongly improves overall survival (*p* < 0.05). After propensity-score matching, Cox regression identified dMMR status as a significant marker of improved overall survival (HR = 0.27, 95%CI 0.09–0.88, *p* = 0.029). **Conclusions**: Overall, our findings suggest that dMMR status is associated with markedly improved survival outcomes in patients after resection of pancreatic and periampullary cancer. Future large-scale studies are needed to further validate this finding.

## 1. Introduction

Pancreatic cancer is the third leading cause of cancer-related death for both men and women in the United States. The disease is often diagnosed at a late stage, when prognosis is poor, and 5-year overall survival rates are only 13% [1,2]. Understanding the oncogenic mutations of pancreatic tumors is imperative upon diagnosis, as specific gene signatures can predict long-term oncologic outcomes and implicate more effective targeted therapies.

DNA mismatch repair (MMR) is a key process that corrects the errors of DNA polymerase during DNA replication, specifically those that occur during the incorporation of complementary base pairs and insertion–deletion mismatches [3]. Mutations in MMR genes, namely MLH1, MSH2, MSH6, and PMS2, can result in defective DNA mismatch repair (also known as MMR-deficiency, [d-MMR]). This deficiency is closely tied to increased tumorigenesis and microsatellite instability (MSI) [4,5]. The unstable genome resulting from d-MMR/MSI becomes an ideal target for immunotherapy, as the resulting increase in mutational burden increases the potential for immunoreactivity from neoantigens.

d-MMR/MSI is an uncommon finding in pancreatic ductal carcinoma (PDAC), occurring in 1–2% of PDACs. However, it has garnered more attention in the pancreatic cancer treatment landscape, as previous studies have indicated that d-MMR tumors may be associated with improved overall and disease-free survival, though large-scale data are limited [6,7,8,9,10,11,12,13]. Recent trials suggest improved responses to immunotherapies, particularly to PD-1 inhibitors such as pembrolizumab [14,15].

In this study, we aim to add to the growing literature to assess the influence of deficient MMR status on oncologic outcomes specifically in patients after resection of PDAC and periampullary adenocarcinomas.

## 2. Materials and Methods

### 2.1. Study Design and Patient Selection

This is a retrospective case–control study based on a prospectively maintained pancreatic surgery database at Thomas Jefferson University Hospital. Patients who underwent pancreatic resection between 2016 and 2021 were identified. Patient records were reviewed for completeness of clinical, histological, and molecular data. Patients with pancreatic ductal adenocarcinoma (N = 342) and periampullary adenocarcinoma (N = 76) were included in the final cohort.

### 2.2. Data Collection

Patient electronic medical records were reviewed, and demographic, perioperative, histologic, molecular, and oncologic outcome data, including next-generation sequencing data, were recorded. Patients with tumors found to have mutations in MLH1, MSH2, MSH6, and PMS2, or an MSI-high status, were categorized as the deficient MMR group (dMMR), while those with wild-type MLH1, MSH2, MSH6, and PMS2 and MSS (microsatellite-stable) status were categorized as the proficient MMR group (pMMR). The primary outcome in this study was overall survival and the secondary outcome was disease-free survival, with survival defined as the time from curative-intent surgery.

### 2.3. Statistical Analysis

Continuous variables are displayed as medians (IQRs) and categorical variables are displayed as counts (%). Comparisons between normally distributed continuous variables were performed using Student’s *t*-test, while non-parametric distribution was compared using the Mann–Whitney test. Categorical parameters were compared using the chi-square test. Kaplan–Meier plots and Cox regression analyses were performed to assess overall and disease-free survival. The Cox model was iteratively optimized by sequential exclusion of statistically insignificant factors (*p* ≥ 0.2) until the achievement of a final optimal model fit (*p* ≤ 0.05) based on minimization of information loss using the Akaike information criterion (AIC). The initial regression models included tumor type, age, histological features, TNM staging, MMR status, and completion of neoadjuvant and adjuvant therapy. Recognizing that the dMMR group in our study was fairly small, and the large set of assessed variables may result in overfitting of the model, a complementary and size-appropriate secondary analysis employed a 1:2 full optimal propensity-score matching (PSM) based on tumor type, TNM staging, and histologic features (perineural invasion and lymphovascular invasion) to compare oncologic outcomes. PSM was performed individually for PDAC and for periampullary cancer patients, ensuring complete matching of the primary pathology. The R statistical software (version 4.2.0) and the “MatchIt”, “survival”, and “survminer” packages, as well as SPSS (version 20.0.1.0), were used for statistical analyses. *p*-value ≤ 0.05 was considered significant.

### 2.4. Ethical Approval

This study was performed at Thomas Jefferson University Hospital (TJUH) and approved by the local institutional review board. The data supporting the findings of this study are available from the corresponding author upon request.

## 3. Results

### 3.1. Cohort Characteristics

A total of 418 pancreatic and periampullary cancer patients were included in the analysis, with 15 patients identified as dMMR. Out of 342 PDAC cases, 7 patients (2%) were found to be dMMR, as were 8 out of 76 periampullary adenocarcinoma patients (10.5%). In addition to the NGS data, immunohistochemistry staining confirming loss of MMR protein expression was available in 6 of the 15 dMMR patients, and sequencing data identifying MMR mutations were available in 11 of the 15 patients (Supplementary Table S1). Pancreaticobiliary subtype was the most common periampullary subtype, identified in 30 (39.5%) patients, followed by the intestinal subtype in 27 patients (35.5%), ampullary subtype in 12 patients (15.8%), and 7 patients with mixed or other subtypes (9.2%). The median ages of the proficient MMR (pMMR) and dMMR patients were 69.2 years (IQR: 69.5–75.3) and 71.2 years (IQR: 60.8–78.7), respectively (P = NS). The male-to-female distribution was similar between the groups (53%:47%, P = NS). Cancer staging, as well as lymphovascular invasion status, were similar between groups, while the pMMR cohort was significantly enriched for PDAC patients and perineural invasion (*p* = 0.002 and *p* = 0.02, respectively), as seen in Table 1. Approximately 70% of patients received perioperative chemotherapy and none of the dMMR patients received immune checkpoint inhibitors during their follow-up period.

### 3.2. Survival Regression Analyses Identify Deficient MMR Status as a Favorable Prognostic Factor

The overall postoperative survival in the study cohort was 31.4 ± 1.8 months, with PDAC patients having a median overall survival of 30.2 ± 2.0 months and periampullary adenocarcinoma patients having a median overall survival of 44.2 ± 10.6 months. PDAC patients with proficient MMR tumors had an overall survival of 30.2 ± 2.0 months while the dMMR group did not reach their median survival (P = NS). Periampullary adenocarcinoma patients with proficient MMR tumors did not have a significant difference in medial overall survival times (65.0 ± 21.0 months vs. 37.3 ± 9.0 months, P = NS). The median disease-free survival (DFS) in the study cohort was 26.3 ± 2.3 months, with PDAC patients having a median overall survival of 22.5 ± 2.2 months and periampullary adenocarcinoma patients having a median DFS of 47.7 ± 9.6 months. PDAC patients with proficient MMR tumors had a DFS of 22.5 ± 2.2 months, while the dMMR group did not reach their median survival (P = NS). Periampullary adenocarcinoma patients with proficient MMR tumors had a median DFS of 47.7 ± 10.4 months, while the dMMR group did not reach their median survival (P = NS).

As the small dMMR group size and large variability limited the interpretability of the Kaplan–Meier results, Cox regression models and propensity-score matching were used to control for potential relevant survival factors. Initial regression models included tumor type (PDAC vs. periampullary), TNM stage, perineural invasion (PNI), lymphovascular invasion (LVI), and the interaction between MMR status and these parameters, along with age and completion of neoadjuvant therapy and adjuvant therapy. Tumor staging and nodal stage were not found to contribute to the output of the models and were removed, as was age in the DFS model, yielding an improved and more significant final model (Table 2).

**Figure 1:**
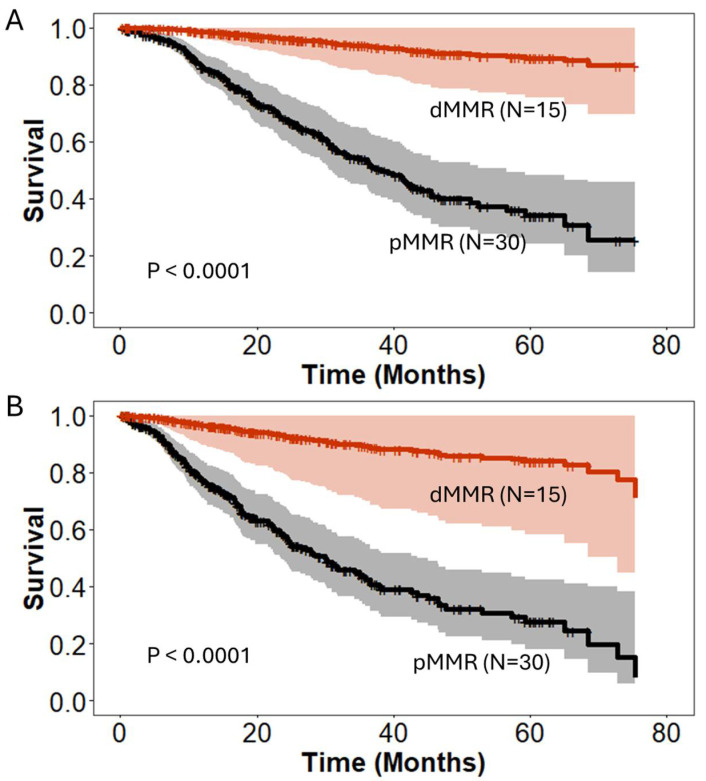
Cox regression models for (**A**) overall survival and (**B**) disease-free survival (N = 418). dMMR—deficient MMR; pMMR—proficient MMR.

The final Cox regression model for overall survival showed that improved survival was individually associated with younger age, tumors lacking lymphovascular invasion, and diagnosis of dMMR status, as seen in Table 2 and Figure 1A. However, the interaction analysis of dMMR with other factors shows that the co-occurrence of dMMR with LVI, PNI, or periampullary cancer was associated with a decrease in the beneficial effect of dMMR. The regression analysis of disease-free survival revealed similar findings, with periampullary tumors, favorable histology lacking lymphovascular or perineural invasion, and dMMR status identified as significant predictors of improved survival (Figure 1B). Similarly to OS analysis, the co-occurrence of dMMR with LVI, PNI, or periampullary cancer was associated with a decrease in the beneficial effect of dMMR.

### 3.3. Propensity-Score Matching Identifies dMMR as a Prognostic Factor for Overall Survival in a Tumor Type-Dependent Manner

In order to improve the robustness of the analysis and decrease variability, a 1:2 optimal fitting propensity-score matching algorithm was used, matching two pMMR patients to every single dMMR patient (Table 3). The resulting groups were fairly evenly matched, without significant differences in presentation and risk factors. Then, a Cox regression was employed to assess the impact of dMMR, tumor type, and their interaction on survival.

**Figure 2:**
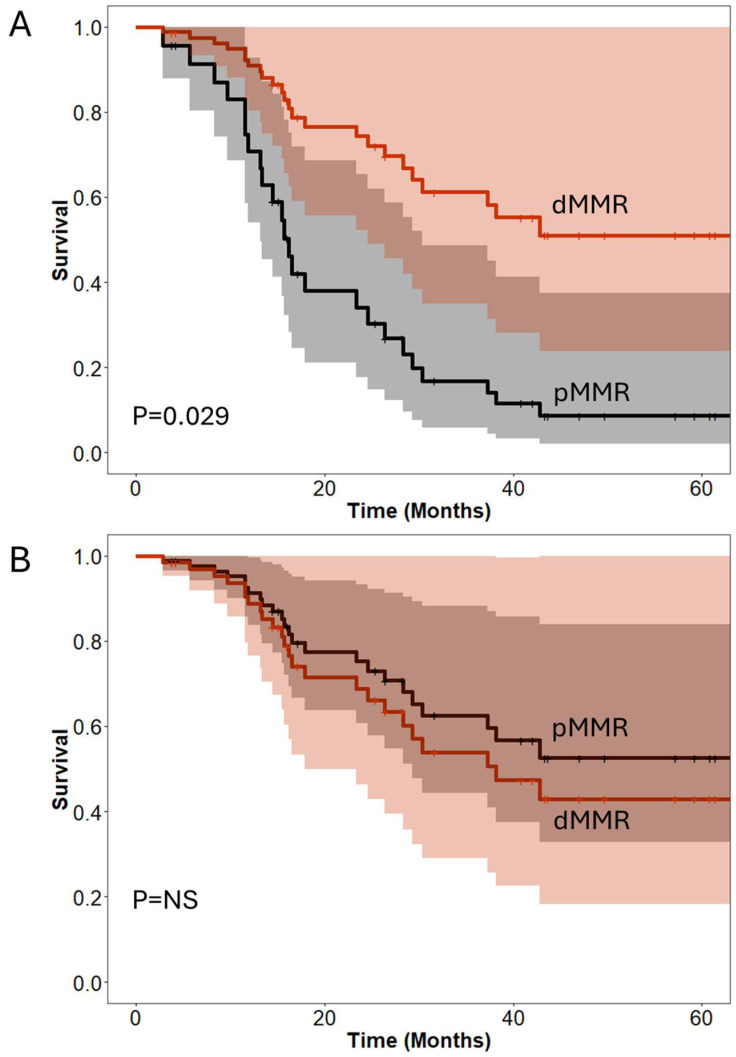
Propensity-score-matched (1:2) and weighted Cox regression model for overall survival analysis in pancreatic ductal adenocarcinoma (**A**) and periampullary cancer patients (**B**), assessing the impact of deficient MMR on the two cancer types (dMMR = 15/pMMR = 30).

Analysis of overall survival (Table 4) showed both dMMR (HR = 0.27, 95% CI 0.09–0.88, *p* = 0.029) and periampullary adenocarcinoma (HR = 0.26, 95% CI 0.12–0.56, *p* = 0.0005) were associated with significantly improved survival, as shown in Figure 2A for PDAC. However, the interaction of both dMMR and periampullary cancers abrogated the effects, resulting in no significant difference in survival in patients with dMMR periampullary cancers. Cox regression analysis of disease-free survival in the matched cohort failed to generate any significant models (Figure 2B, P = NS). In order to account for the time offset from diagnosis in patients who have received neoadjuvant therapy, we also conducted a supplementary survival analysis based on time from initial diagnosis (Supplementary Table S2, Supplementary Figure S1). This resulted in fairly similar effect estimation of dMMR status (HR 0.25, 95% CI 0.09–0.72, *p* = 0.010).

## 4. Discussion

Mismatch repair deficiency, particularly in the context of immune checkpoint inhibitor therapy, is associated with improved outcomes in multiple cancer types, such as colorectal, endometrial, gastric, small intestine, and urothelial cancers [16]. Though relatively rare in PDAC, MMR mutations have been suggested to increase patient survival. In this study, we have aimed to explore the impact of MMR mutation on the survival outcomes of patients with resected PDAC and periampullary adenocarcinoma.

Pancreatic cancer is an aggressive GI malignancy, with notable resistance to standard chemotherapy and radiotherapy. Furthermore, since most pancreatic cancers present with a relatively low tumor mutational burden, these tumors are fairly resistant to current immunotherapy as well. However, about 1–2% of cases of pancreatic ductal adenocarcinoma exhibit microsatellite instability, defects in mismatch repair (MMR), and high tumor mutational burden. This specific subgroup of patients tends to show an improved response to immunotherapies [17].

While there are data supporting a beneficial impact of dMMR status on overall survival in advanced-stage pancreatic cancer, there are limited data on the impact of dMMR on early-stage, resected PDAC [16]. Using our prospectively maintained surgical database, we assessed outcomes in 418 patients who underwent pancreatic resection due to PDAC or periampullary cancer in the years 2016–2021. We initially used logistic regression analyses to identify d-MMR status as a major factor associated with improved overall and disease-free survival after resection. However, as the interpretation of the regression results is limited by the small number of d-MMR patients and the risks of model overfitting, we advanced to include a propensity-score matching to provide a more appropriate survival analysis. This was a tumor type-specific propensity-score matching (for PDAC and periampullary ca. each) to better control the groups for age, TNM staging, and high-risk histologic features (perineural invasion and lymphovascular invasion). The results of our Cox regression analysis suggest that d-MMR/MSI is associated with improved disease-free and overall survival in early-stage PDAC and periampullary adenocarcinoma. However, the beneficial effects of d-MMR status seem to be markedly greater in PDAC compared to periampullary cancers, as the interaction analysis in Table 2 shows d-MMR status has a 25-fold lesser impact on OS in periampullary cancer and a 10-fold lesser impact on DFS in periampullary cancers. Our propensity-score-matched regression analysis revealed a significant improvement in overall survival in patients with PDAC but failed to show significant outcome differences in periampullary cancers or in disease-free survival. This was also evident when we corrected for possible bias from neoadjuvant therapy by analyzing survival time from diagnosis.

In 1998, Goggins et al. examined the influence of d-MMR/MSI status on prognosis in PDAC by closely analyzing and classifying pancreatic carcinomas with microsatellite instability. Several histopathologic and clinical parameters were assessed in tumor samples from 82 patients, only three of which were classified as MSI. The results suggested that patients with MSI may potentially exhibit improved survival; however, the study’s small cohort size necessitated further investigation [6]. Shortly thereafter, Yamomoto and colleagues also assessed survival in patients with PDAC. Their study included 100 PDAC patients (32% early-stage, 64% locally advanced/metastatic), 13 of whom were diagnosed as d-MMR/MSI-H (13%). Survival analyses also revealed improved survival, further indicating that mutations in MMR contribute to improved prognosis [8]. Similar findings were reported by Nakata and colleagues, looking at a combined cohort of early-stage and metastatic patients [9].

More recently, d-MMR/MSI has been examined in the context of immunotherapy, as tumors with mismatch repair deficiency have been shown to exhibit improved responses to PD-1 blockade therapies [18]. This indeed holds true for pancreatic carcinomas [13,19]. Hu and colleagues not only found that patients with d-MMR pancreatic carcinoma exhibited favorable therapeutic outcomes, but also suggested that the natural history of PDAC with mismatch repair deficiency was improved compared to that of PDAC proficient mismatch repair [13]. Additionally, Grant et al. conducted a study on resectable dMMR pancreatic cancer cases and found that these patients had longer overall survival after surgery. This group also reported that dMMR tumors were less likely to have mutations in traditional pancreatic cancer markers such as KRAS and SMAD4, but rather showed mutations in JAK1 and ACV2RA which correspond with microsatellite instability [7].

National Comprehensive Cancer Network guidelines support the administration of PD-1 blockade therapy for patients with unresectable or metastatic MSI-H or dMMR solid tumors that have progressed despite previous treatments and who have no recommended alternative treatment [20]. These recommendations are based in part on the KEYNOTE-158 trial, a phase II clinical trial for pembrolizumab that included a cohort of 233 patients with various types of previously treated advanced non-colorectal MSI-H/dMMR cancer, 22 of whom had pancreatic cancer. In the pancreatic cancer group, an objective response rate of 18.2%, a median progression-free survival of 2.1 months, and a median overall survival of 4 months were reported [21]. Interestingly, a recent study conducted by Coston et al. in 2023 suggested that immune checkpoint inhibitors such as pembrolizumab may be superior in effect over standard cytotoxic chemotherapy across multiple stages of cancer progression. In their cohort of 32 patients with dMMR/MSI-H solid pancreatic tumors, they found that pembrolizumab had an overall response rate of 75% and a disease control rate of 90%; in comparison, cytotoxic chemotherapy had a 30% overall response rate and a disease control rate of 60% [22].

There are several theories as to the mechanism through which d-MMR leads to improved survival. One proposed mechanism of the improved prognosis is that the loss of function mutation of MMR proteins such as MSH2, MLH1, MSH6, and PMS2 causes an increased tumor mutational burden [23]. As a result of deficient MMR mechanisms, cancer cells produce more neoantigens which can be recognized by the immune system as foreign, thus potentially increasing efficacy of immunotherapy [24]. Other studies have shown that MMR-deficient cancers have increased cytotoxic T cell infiltration and higher tumor checkpoint protein expression, which are both correlated with better prognosis and response to immunotherapy [25]. Our results support the narrative that PDAC with d-MMR, though rare, has a uniquely different biology and outcome. This also raises the question of the possible utility of immune checkpoint inhibitors in pancreatic cancer in the neoadjuvant and adjuvant setting. As pembrolizumab has shown a striking response in the treatment of other early-stage cancers [26,27,28,29], we can hope a similar response would be achieved in the pancreatic and periampullary cancer patient group.

It is important to note several limitations of our study. First, this is a retrospective study, and as such it is limited in attributing cause and effect. This is especially relevant when considering the histological features and genetic landscape of dMMR tumors, which are very different than those of pMMR tumors: dMMR tumors have significantly higher rates of medullary and colloid/mucinous histology [6,7]. This is coupled with significantly higher rates of wild-type KRAS and wild-type P53, occurring in up to two-thirds of patients [6]. Additionally, periampullary cancer is an umbrella term that covers multiple histological subtypes (pancreaticobiliary, intestinal, ampullary, and mixed types), each associated with different oncological outcomes and therapy response rates. Due to the limited size of our cohort, we were unable to fully account for the specific impact of these individual subtypes. Furthermore, due to the rarity of d-MMR/MSI in pancreatic cancers, cohort sizes in most related studies are quite small and contribute to limitations in study designs. In our cohort, only 3.5% of all patients had tumors with MMR mutations (2% for PDAC). While this greatly limits the interpretation of overly dimensional regression, this was addressed in part by the employment of propensity-score matching for our survival analysis. Future studies might utilize a larger database with many more patients to validate our findings.

## 5. Conclusions

Overall, the results of this study indicate dMMR status to be independently associated with markedly improved survival outcomes in patients post resection of pancreatic cancer and potentially periampullary cancer as well. These findings also suggest and support the postulate that PD-1 checkpoint inhibition may play a role in either the adjuvant or neoadjuvant treatment of patients with dMMR tumors. Future large-scale clinical studies are needed to further validate this finding and test this potential application.

## Figures and Tables

**Table 1:** Cohort demographics (N = 418).

N = 418		Proficient MMR Status (N = 403)	Deficient MMR Status (N = 15)	*p*-Value
Age, Median (IQR)		69.2 (69.5–75.3)	71.2 (60.8–78.7)	0.422
Sex	Male	189 (46.9)	7 (46.7)	0.598
	Female	214 (53.1)	8 (53.3)	
Histology	PDAC	335 (83.1)	7 (46.7)	0.002
	Periampullary Ca.	68 (16.9)	8 (53.3)	
T	0	2 (0.5)		0.116
	1	62 (15.5)	1 (6.7)	
	2	198 (47.1)	4 (26.7)	
	3	133 (33.2)	10 (66.7)	
	4	15 (3.7)		
	Missing/Unknown	2		
N	0	148 (36.8)	6 (40)	0.741
	1	167 (41.5)	7 (46.7)	
	2	87 (21.6)	2 (13.3)	
	Missing/Unknown	1		
Perineural invasion		334 (84.3)	9 (60)	0.024
	Missing/Undetermined	7		
Lymphovascular invasion		192 (48.6)	9 (60)	0.274
	Missing/Undetermined	8		
Involved resection margins (R1)		39 (9.7)	0 (0)	0.379
	Missing/Undetermined	1		
Neoadjuvant chemotherapy		85 (22)	2 (13.3)	0.537
	Missing/Undetermined	28		
Neoadjuvant radiotherapy		21 (6.8)	0 (0)	0.625
	Missing/Undetermined	95	4	
Adjuvant chemotherapy		244 (64.7)	8 (53.3)	0.413
	Missing/Undetermined	26		
Adjuvant radiotherapy		99 (40.1)	2 (28.6)	0.706
	Missing/Undetermined	156	8	
Any Perioperative Chemotherapy		276 (75.2)	9 (60)	0.223
	Missing/Undetermined	36		

MMR—mismatch repair; PDAC—pancreatic ductal adenocarcinoma; IQR—interquartile range.

**Table 2:** Cox regression models for overall survival and disease-free survival (N = 418). PDAC—pancreatic ductal adenocarcinoma.

		Overall Survival		Disease-Free Survival	
		HR (95% CI)	*p*-Value	HR (95% CI)	*p*-Value
Age at diagnosis		1.02 (1–1.04)	0.017	-	-
Histology					
PDAC		Reference		Reference	
Periampullary Ca.		0.71 (0.42–1.21)	0.211	0.55 (0.36–0.83)	0.005
Perineural invasion		1.77 (1.07–2.93)	0.045	1.80 (1.08–2.99)	0.026
Lymphovascular invasion		1.59 (1.15–2.20)	0.0003	1.59 (1.18–2.15)	0.005
Neoadjuvant therapy		1.43 (1.03–1.99)	0.033	1.39 (0.92–2.09)	0.11
Adjuvant therapy		-	-	-	-
dMMR		0.01 (0.002–0.05)	<0.0001	1.3 × 10^−8^ (1.5 × 10^−9^–1.0 × 10^−7^)	<0.0001
Interactions	dMMR: Periampullary Ca.	23.87 (5.26–108.37)	<0.0001	9.78 (2.45–3.91)	<0.0001
	dMMR: Perineural invasion	10.69 (4.07–28.09)	<0.0001	1.1 × 10^7^ (2.5 × 10^6^–4.6 × 10^7^)	<0.0001
	dMMR: Lymphovascular invasion	6.29 (2.53–15.67)	0.0001	6.14 (0.94–4.03)	0.0589

HR—hazard ratio; CI—confidence interval; dMMR—deficient mismatch repair.

**Table 3:** Propensity-score-matched cohort demographics (N = 45), optimal 1:2 matching.

N = 45		Proficient MMR Status (N = 30)	Deficient MMR Status (N = 15)	*p*-Value
Age, Median (IQR)		71 (61–78.25)	71 (60–78)	0.952
Sex	Male	13 (43.3)	7 (46.7)	0.54
	Female	17 (56.7)	8 (53.3)	
Histology	PDAC	14 (46.7)	7 (46.7)	0.623
	Periampullary Ca.	16 (43.3)	8 (53.3)	
Periampullary Subtype (N = 16)	Pancreaticobiliary	8 (50%)	3 (37.5%)	0.369
	Intestinal	5 (31.2%)	1 (12.5%)	
	Ampullary	2 (12.5%)	3 (37.5%)	
	Mixed/Other	1 (6.3%)	1 (12.5%)	
T	1	2 (6.7)	1 (6.7)	0.877
	2	6 (20.0)	4 (26.7)	
	3	22 (73.3)	10 (66.7)	
N	0	11 (36.7)	6 (40)	0.974
	1	15 (50.0)	7 (46.7)	
	2	4 (13.3)	2 (13.3)	
Perineural invasion	No invasion	11 (36.7)	6 (40)	0.539
	Invasion	19 (63.3)	9 (60)	
Lymphovascular invasion	No invasion	13 (43.3)	6 (40)	0.545
	Invasion	17 (56.7)	9 (60)	
Neoadjuvant chemotherapy		4 (13.8)	2 (13.3)	1.0
Missing/Undetermined		1		
Adjuvant chemotherapy		16 (55.2)	8 (53.3)	1.0
Missing/Undetermined		1		

MMR—mismatch repair; PDAC—pancreatic ductal adenocarcinoma; IQR—interquartile range.

**Table 4:** Cox regression analysis of mismatch repair status and tumor type as predictors of overall survival in a propensity-score-matched cohort (dMMR = 15/pMMR = 30).

N = 45	HR (95% CI)	*p*-Value
Histology		
PDAC	Reference	-
Periampullary Ca.	0.26 (0.12–0.56)	0.0005
dMMR	0.27 (0.09–0.88)	0.029
dMMR:Periampullary Ca.	4.79 (1.0–22.9)	0.050

PDAC—pancreatic ductal adenocarcinoma; HR—hazard ratio; CI—confidence interval; dMMR—deficient mismatch repair.

## Data Availability

The data presented in this study are available upon request from the corresponding author. The study data are not publicly available because it is part of a large prospectively maintained database containing genetic data.

## Supplementary Materials

The following supporting information can be downloaded at https://www.mdpi.com/article/10.3390/jcm13206185/s1. Table S1. Immunohestochemistry (IHC) and next generation sequencing (NGS) findings of dMMR patients (N = 15). Table S2. Cox regression analysis of mismatch repair status and tumor type as predictors of overall survival (time from diagnosis) in a propensity-score matched cohort (dMMR = 15/Pmmr = 30). Figure S1. Propensity-score matched (1:2) and weighted Cox regression model for overall survival (time from diagnosis) analysis in pancreatic ductal adenocarcinoma (A) and periampullary cancer patients (B), assessing impact of deficient MMR with the two cancer types (dMMR = 15/pMMR = 30).
