# Supplementary material for: Assessing Influence of Mismatch Repair Mutations on Survival in Patients After Resection of Pancreatic Ductal and Periampullary Adenocarcinoma
Source: J Clin Med. 2024 Oct 17;13(20):6185. doi: 10.3390/jcm13206185 (PMC11508431; doi:10.3390/jcm13206185)
Supplement: Supplementary file 1 [file jcm-13-06185-s001.zip › jcm-3140287-supplementary.pdf]

Supplementary Table S1. Immunohestochemistry (IHC) and next generation sequencing (NGS) findings of dMMR patients (N=15).

Case#	IHC	NGS
1	Loss of MLH1/PMS2	Not avialable
2	Loss of MLH1/PMS2	Not avialable
3	Loss of MLH1/PMS2	Not avialable
4	Loss of MSH2/MSH6	MSH2 - deletion of exons 7,9
5	Loss of PMS2,<br>MLH1 equivocal	Not avialable
6	Loss of MSH6	Genetic MLH1 Q701*
7	Equivocal MSH6	PMS deletion of exon 13-14
8	No loss	MLH1 p.L228M
9	No loss	Germline: PMS2 c873delT<br>Somatic: PMS2 5'UTR_3'UTR deletion;
10	No loss	PMS D493N
11	No loss	PMS2 truncation of exon6
12	No loss	PMS2 truncation of exon6
13	No loss	MSH6 Mutation p.K1358fs*2 (Frame-Shift, Exon 10)
14	IHC not available	MSH6 Mutation p.K1358fs (Frame-Shift, Exon 10)
15	IHC not available	PMS2 H479Q

Supplementary Table S2. Cox regression analysis of mismatch repair status and tumor type as predictors of overall survival (time from diagnosis) in a propensity-score matched cohort (dMMR=15/pMMR=30).

N=45	HR (95% CI)	P-Value
Histology		
PDAC	Reference	-
Periampullary Ca.	0.26 (0.12-0.56)	0.0004
dMMR	0.25 (0.09-0.72)	0.010
dMMR:Periampullary Ca.	4.79 (1.0 – 22.9)	0.028

PDAC – pancreatic ductal adenocarcinoma; HR – hazard ratio; CI – confidence interval; dMMR – deficient mismatch repair.

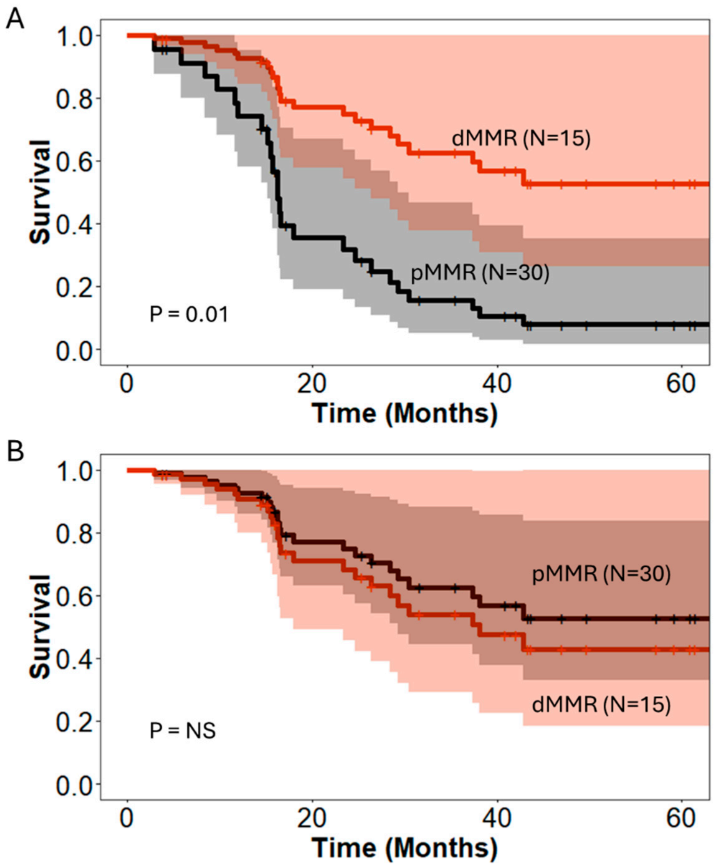

Supplementary Figure S1. Propensity-score matched (1:2) and weighted Cox regression model for overall survival (time from diagnosis) analysis in pancreatic ductal adenocarcinoma (A) and periampullary cancer patients (B), assessing impact of deficient MMR with the two cancer types (dMMR=15/pMMR=30).
